# Gene expression profiling of perineural invasion in head and neck cutaneous squamous cell carcinoma

**DOI:** 10.1038/s41598-021-92335-4

**Published:** 2021-06-23

**Authors:** Timothy J. Eviston, Elahe Minaei, Simon A. Mueller, Navid Ahmadi, Bruce Ashford, Jonathan R. Clark, Nicholas West, Ping Zhang, Ruta Gupta, Marie Ranson

**Affiliations:** 1 The Sydney Head and Neck Cancer Institute, Chris O’Brien Lifehouse, Sydney, NSW Australia; 2 School of Chemistry and Molecular Bioscience, University of Wollongong, Wollongong, NSW Australia; 3 Illawarra Health and Medical Research Institute (IHMRI), Wollongong, NSW Australia; 4 Centre for Oncology Education and Research Translation (CONCERT), Sydney, NSW Australia; 5 Department of Oto-Rhino-Laryngology, Head and Neck Surgery, Inselspital, Bern University Hospital, University of Bern, Bern, Switzerland; 6 Faculty of Medicine, University of New South Wales, Sydney, NSW Australia; 7 Illawarra and Shoalhaven Local Health District (ISLHD), Wollongong, NSW Australia; 8 School of Medicine, University of Wollongong, Wollongong, NSW Australia; 9 Royal Prince Alfred Institute of Academic Surgery, Sydney Local Health District, Sydney, Australia; 10 Sydney Medical School, Faculty of Medicine and Health Sciences, The University of Sydney, Sydney, Australia; 11 Systems Biology and Data Science, Griffith Systems Biology Centre, Menzies Health Institute Queensland, Griffith University, Southport, QLD Australia; 12 Tissue Pathology and Diagnostic Oncology, Royal Prince Alfred Hospital, NSW Health Pathology, Sydney, Australia

**Keywords:** Squamous cell carcinoma, Cancer genomics

## Abstract

Perineural invasion (PNI) is frequently associated with aggressive clinical behaviour in head and neck cutaneous squamous cell carcinoma (HNcSCC) leading to local recurrence and treatment failure. This study evaluates the gene expression profiles of HNcSCC with PNI using a differential expression analysis approach and constructs a tailored gene panel for sensitivity and specificity analysis. 45 cases of HNcSCC were stratified into three groups (Extensive, Focal and Non PNI) based on predefined clinicopathological criteria. Here we show HNcSCC with extensive PNI demonstrates significant up- and down-regulation of 144 genes associated with extracellular matrix interactions, epithelial to mesenchymal transition, cell adhesion, cellular motility, angiogenesis, and cellular differentiation. Gene expression of focal and non PNI cohorts were indistinguishable and were combined for further analyses. There is clinicopathological correlation between gene expression analysis findings and disease behaviour and a tailored panel of 10 genes was able to identify extensive PNI with 96% sensitivity and 95% specificity.

## Introduction

Cutaneous squamous cell carcinoma (cSCC) is the second most common malignancy and frequently occurs in the head and neck^1^. This cancer is treated by a diverse range of clinicians including surgeons, primary care physicians and dermatologists. Although most cSCC are adequately treated with local treatment, a small but important minority follow a more aggressive course which can result in locoregional failure and/or metastatic disease and subsequent incurable disease. Accurate pathological assessment of adverse features such as perineural invasion (PNI) in cSCC is critical to decision making, yet the differentiation between clinically significant and incidental PNI remains a challenge.

Perineural invasion (PNI) is an established adverse prognostic factor in multiple malignancies including head and neck cutaneous squamous cell carcinoma (HNcSCC)^2–4^. PNI is defined as tumor infiltration of the perineurium along at least one third of the circumference of the nerve in any of the three layers^2^. The American Joint Commission on Cancer (AJCC) has recognised PNI of a nerve > 0.1 mm as a high risk factor for cutaneous SCC since 2010 due to the increased risk of local recurrence, metastasis and disease specific death^5,6^. The presence of PNI is usually an indication for more aggressive surgery and/or adjuvant radiotherapy^3,7^.

In the head and neck, PNI can extend proximally along cranial nerves to involve the skull base and ultimately the brain, referred to as perineural spread (PNS)^7^. Clinically this can present as altered sensation and/or loss of function of involved nerves^8^. While nearly 14–16% of HNcSCC demonstrate PNI, PNS is seen in less than 5% of cases^9^. Greater understanding of the genetic factors and underlying mechanisms that drive the development of clinically aggressive PNI in HNcSCC is important to determine prognosis, identify new therapeutic targets and guide decision making with regard to treatment escalation, operative strategy and radiotherapy planning.

The primary aim of this study was thus to investigate whether gene expression profiling could distinguish HNcSCCs with extensive PNI from those without extensive PNI (focal or non-PNI). The secondary aim was to construct a PNI-specific gene signature based on its ability to predict extensive PNI in HNcSCCs. With further validation, this panel could assist in the objective determination of clinically significant PNI and aid the personalisation of cancer treatment decisions.

## Results

### Clinical and demographic characteristics

Clinical and demographic data are shown in Table 1. The final cohort of 45 patients (6 females and 39 males) were classified into three groups based on pathological criteria (Non-PNI, Focal-PNI, EXT-PNI). Patient demographics (mean age and sex distribution) and tumor characteristics [mean tumor width (33–34 mm) and mean thickness (10–12 mm)] were similar between groups. Consistent with the gene expression profiling (described below), the Non-PNI and Focal-PNI groups were combined for analysis. The EXT-PNI group had a higher number of nose and midface primaries (p < 0.01), a higher proportion were recurrent tumors (p < 0.01) and higher disease specific mortality (p < 0.001). There was no difference in the rate of lymph node metastases (p = 0.31). The EXT-PNI group had higher AJCC T-category and overall stage, as any cSCC with invasion of named nerves is classified at-least T3 and stage III.

**Table 1:** Demographic and clinical data for the 45 samples included in the study.

Parameter	Non-PNI (n = 9)	Focal (n = 11)	Extensive (n = 25)	*p*-value^1^
Mean age, years (range)	69.6	70.8	71.0	NS (p = 0.46)
**Sex, n (%)**				
Female	1	1	4	NS (p = 0.31)
Male	8	10	21	
**Location of primary tumor, n (%)**				
Scalp	4	1	8	p < 0.01*
Ear and temple	5	5	8	
Nose and midface	0	2	8	
Lip	0	2	1	
Neck	0	1	0	
**Size of primary tumor**				
Average tumor width (mm)	33	34	33	NS (p = 0.98)
Average tumor thickness (mm)	10	12	11	NS (p = 0.89)
**T-stage, n (%)**				
1	3	1	0	p < 0.0001*
2	1	6	0	
3	4	4	23	
4	1	0	2	
**N-stage, n (%)**				
0	7	8	21	NS (p = 0.31)
1	0	1	2	
2	0	2	2	
3	2	0	0	
**AJCC overall stage, n (%)**				
I	3	1	0	p < 0.0001*
II	1	4	0	
III	3	4	22	
IV	2	2	3	
**Recurrence**				
Yes	3	3	14	p < 0.01*
No	6	8	11	
**Outcome**				
Alive, no evidence of disease	6	8	9	p < 0.0001*
Dead of disease	1	1	7	
Alive with disease	0	1	6	
Dead of other causes	2	1	3	
Average follow up (months)	50	43	23	

The difference in recurrence and mortality for EXT-PNI compared to Focal and Non-PNI highlight the distinct clinical course that extensive PNI tumors follow compared to focal and non PNI tumors. The preponderance of midface and ear and temple cSCCs in the EXT-PNI group has been demonstrated in other studies with the trigeminal nerve (CNV) and facial nerve (CNVII) being the most common nerves affected^3^.

### Tumor RNA profiling

**Figure 1:**
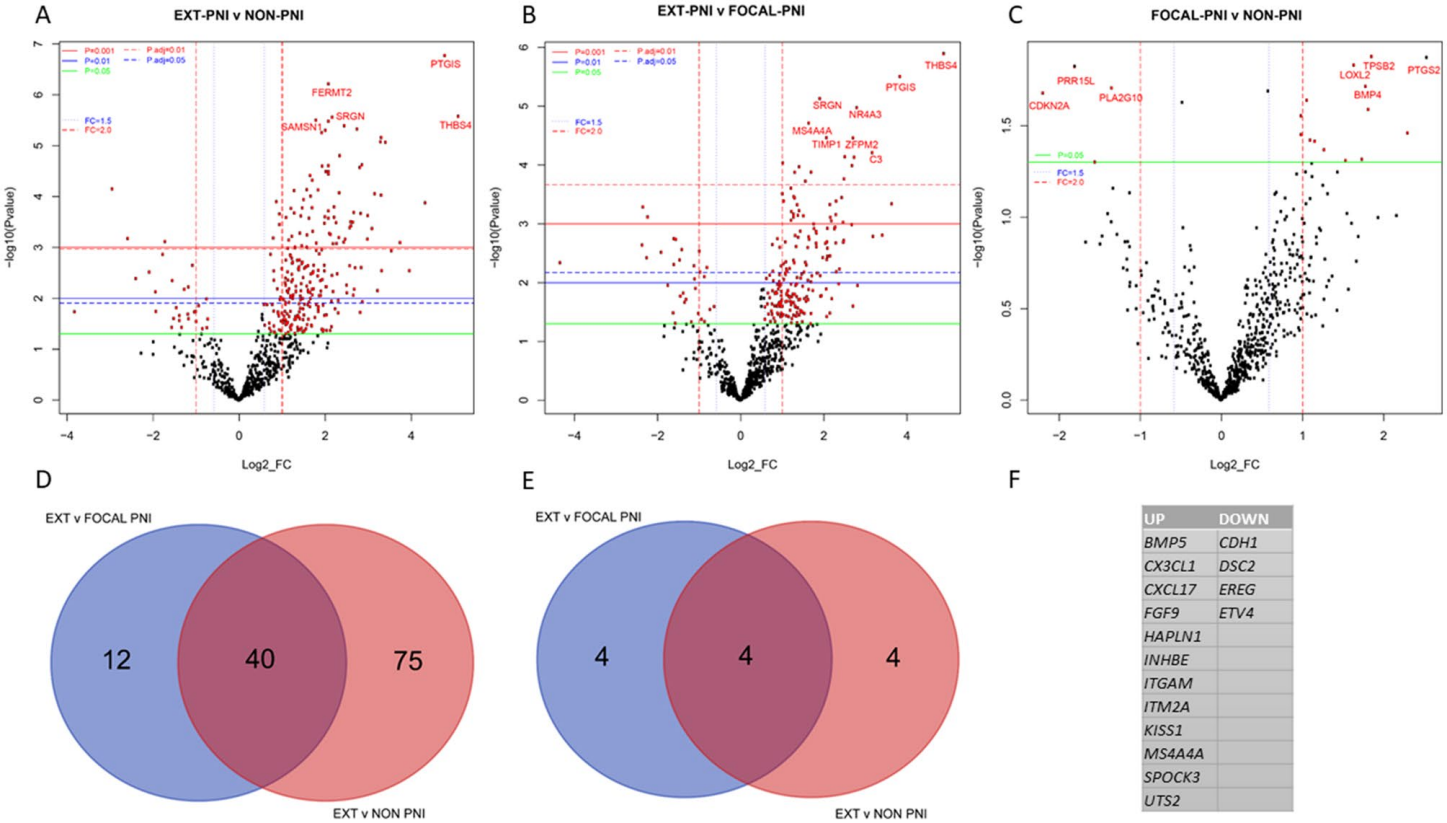
Differential gene expression between PNI cohorts. (**A**–**C**) Volcano plots showing each gene’s − log10 (*p*-value) and log2 fold change of (**A**) EXT-PNI versus Non-PNI, (**B**) EXT-PNI versus Focal-PNI, and (**C**) Focal-PNI versus Non-PNI cohorts. Genes that fall at the top and to either side of the plot are the most significantly DEGs. Dashed colored horizontal lines indicate adjusted *p*-value thresholds as shown. Solid colored horizontal lines indicate unadjusted *p*-value thresholds as shown. Dashed coloured vertical lines indicate fold change of ± 1.5 or 2 × (FC = 1.5 and FC = 2.0). The most statistically significant genes are labeled. (**D**,**E**) Venn diagrams showing DEG with (**D**) ≥ 3 or (**E**) ≤ − 3-fold change between cohorts as shown and adjusted *p*-values of < 0.01. The number of DEG for each pairwise comparison is indicated in the circles. Blue circles; DEG between EXT vs. Focal-PNI. Orange circles; DEG between EXT vs. Non-PNI. The overlap between the circles (purple) shows shared DEG genes between comparisons. (**F**) Significant up and down DEGs unique to EXT-PNI versus Focal-PNI comparison.

**Figure 2:**
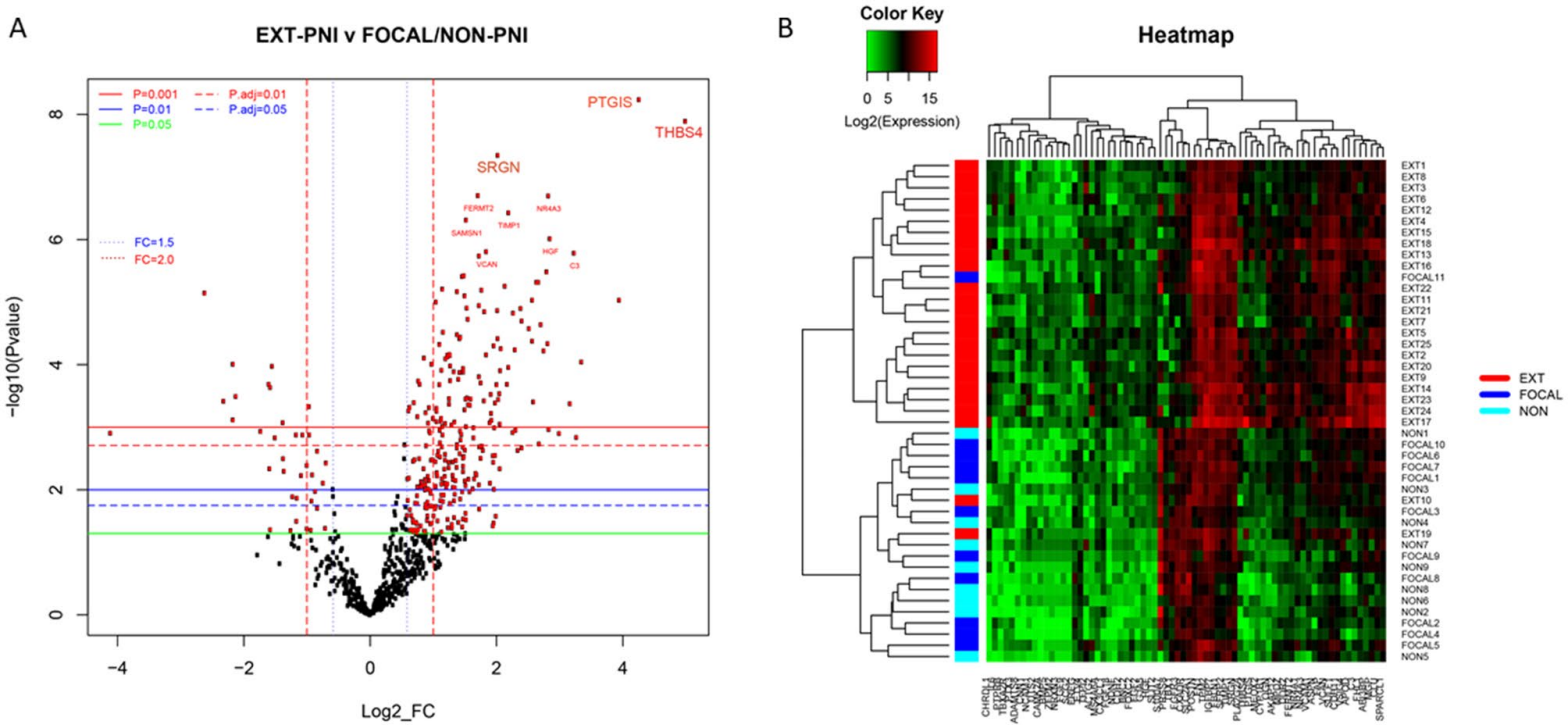
Differential gene expression between EXT PNI versus focal/non PNI cohorts. (**A**) Volcano plot showing each genes − log10 (*p*-value) and log2 fold change (FC) of EXT-PNI compared to combined Focal/Non-PNI cohorts. Highly statistically significant and differentially expressed genes fall at the top or to either side of the plot, respectively. Colored horizontal lines indicate various unadjusted and adjusted *p*-value thresholds. Vertical lines indicate FC of ± 1.5 or 2 × (FC = 1.5 and FC = 2.0). The most statistically significant genes are specifically labeled. (**B**) Heatmap of the normalized data, scaled to give all genes equal variance, generated via unsupervised clustering based on the highest ranking differentially expressed genes between EXT and Focal/Non-PNI cohorts with fold change > 3, < − 3 and adjusted *p*-value < 0.01. Green represents low expression, Red represents high expression.

Significant upregulated differential gene expression was observed between the EXT-PNI versus Non-PNI or Focal-PNI groups, with few significant downregulated genes (Fig. 1A,B). There were no statistically significant DEGs between Focal-PNI versus Non-PNI cohorts (Fig. 1C; see also Supplementary Datafile 1). Indeed, many of the significant DEGs with fold-change ≥ 3 or < − 3 between the EXT-PNI versus Focal-PNI and EXT-PNI versus Non-PNI comparisons were shared (40/127 for upregulated DEGs; 4/8 for downregulated DEGs) (Fig. 1D,E), demonstrating substantial overlap between the Focal-PNI and Non-PNI cohorts. There were, however, 12 upregulated and four downregulated genes unique to the EXT-PNI versus Focal-PNI (Fig. 1F). Given their similarity, Focal-PNI and Non-PNI were combined (Focal/Non-PNI; n = 20) for re-analysis (Fig. 2A) demonstrating 144 DEGs with > 2- or < − 2-fold change and 70 DEGs with > 3 or < − 3-fold change at adjusted *p*-values < 0.01 (Supplementary Table 1). Unsupervised hierarchical clustering analysis based on these 70 DEGs with at least threefold change (bolded genes in Supplementary Table 1) confirmed that the EXT-PNI cohort segregated from the Focal-PNI and Non-PNI cohorts, when separated (Fig. 2B) or combined (Supplementary Fig. 1).

### Neural tissue quantification

The amount of neural tissue included in the samples could confound differences observed between groups. This was quantified on histopathologic review and ranged from 0.5 to 80%. Stratification of the EXT-PNI cohort based on low (< 10%) versus high (> 10%) nerve proportion (Supplementary Table 2) did not reveal significant DEGs at adjusted *p*-value < 0.05 (Supplementary Table 3). Furthermore, a comparison of DEGs between EXT-PNI with only low nerve proportion with the Focal/Non-PNI cohort gave similar results to the total EXT-PNI cohort comparison (data not shown) further confirming that nerve proportion is not a confounder.

### Sensitivity and specificity analysis

**Figure 3:**
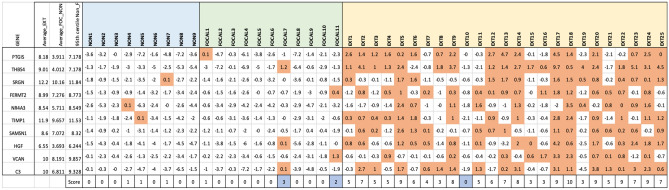
Sensitivity and specificity analysis. Gene panel based on the top 10 most significant genes by adjusted *p*-value. The 95th centile is calculated for each gene using the outputs of Non and Focal-PNI samples. The 95th centile score is subtracted from each sample. Fields that are positive (i.e. > 95th centile) are given a score of 1 and are shaded orange. The total score is then calculated across all 10 genes to give a combined score out of 10. Outliers (shaded blue) are determined as any Non or Focal-PNI samples with a score of > 1, or Extensive PNI with a Score < 2.

**Figure 4:**
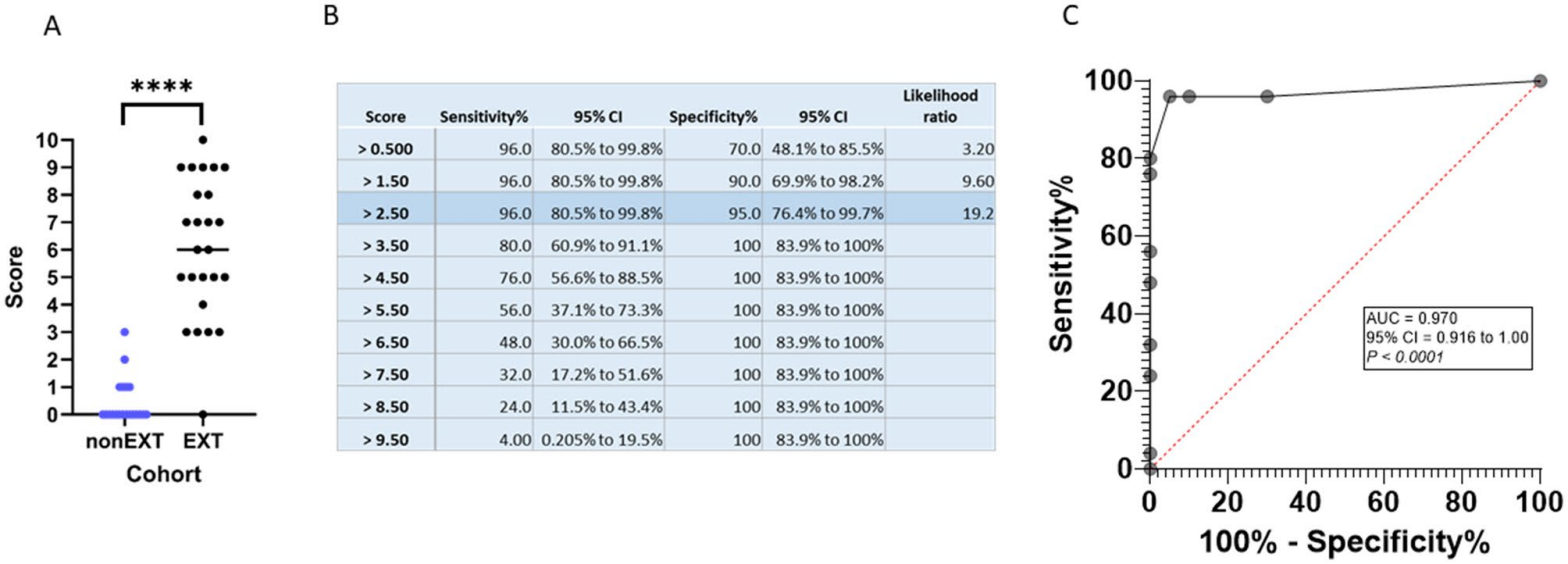
Sensitivity and Specificity analysis. (**A**) Plot of scores derived for Focal-PNI and Non-PNI specimens combined (nonEXT) versus EXT-PNI specimens from the data shown in Fig. 3, (**B**) data table showing sensitivity and specificity at various cut-off scores, and (**C**) the receiver operating characteristic (ROC) curve. AUC and sensitivity and specificity were analyzed and ROC curve generated using the ROC curve function on GraphPad Prism (ver 8.4.3). ****p < 0.0001.

The application of a gene score using a 10 gene panel (Fig. 3) was found to be highly discriminatory (Fig. 4). The cut-off gene score for Focal/Non-PNI samples was ≤ 2 compared to ≥ 3 for the EXT PNI samples (Fig. 3). Using a threshold of 2.5, the gene score was found to have 96% sensitivity, 95% specificity and 97% probability of correctly predicting EXT-PNI (AUC 0.97, p < 0.0001; Fig. 4). False positives included two Focal-PNI samples (FOCAL-7, FOCAL-11). False negatives included one EXT-PNI sample (EXT-10). These outliers match those found using unsupervised clustering described above with the addition of FOCAL-7.

On investigation of Focal-PNI outliers (FOCAL-11 and FOCAL-7) that grouped with the EXT-PNI specimens on heatmap analysis (Fig. 2B) or with score > 2 (Fig. 3) followed similar clinical paths to the EXT-PNI cohort, evidenced by early local recurrence and subsequent incurable disease. FOCAL-11 was a 63 year old male who had radical excision for a large (47 mm) temporo-parietal HNcSCC and adjuvant radiation for a positive deep margin. Within 2 years the patient had widespread local recurrence including intracranial PNS which was deemed irresectable and he succumbed to disease. FOCAL-7 was a 65 year old male with a 53 mm T3N2 cheek HNcSCC. Despite aggressive surgical resection including orbital exenteration, the patient locally recurred within 2 years and subsequently developed intracranial extension at the site of his previous orbital exenteration. The clinical outcomes of these tumors suggest that the biological behavior was more consistent with the expression analysis rather than the original histology.

In contrast, the two EXT-PNI patients (EXT-10 and EXT-19) that grouped with the Focal/Non-PNI cohort on heatmap or score analyses had a clinically aggressive course. EXT-10 was a 84 year old male with a large (50 mm) postauricular cSCC with widespread local invasion extending to the stylomastoid foramen with multiple foci of PNI. The patient died from local recurrence within the first year following treatment. EXT-19 was a 38 year old male with a lip cSCC who had multiple local recurrences in the submental region including soft tissue deposits despite surgery and concurrent chemoradiation. He died 25 months after the initial tumor resection.

### Pathways analyses

**Figure 5:**
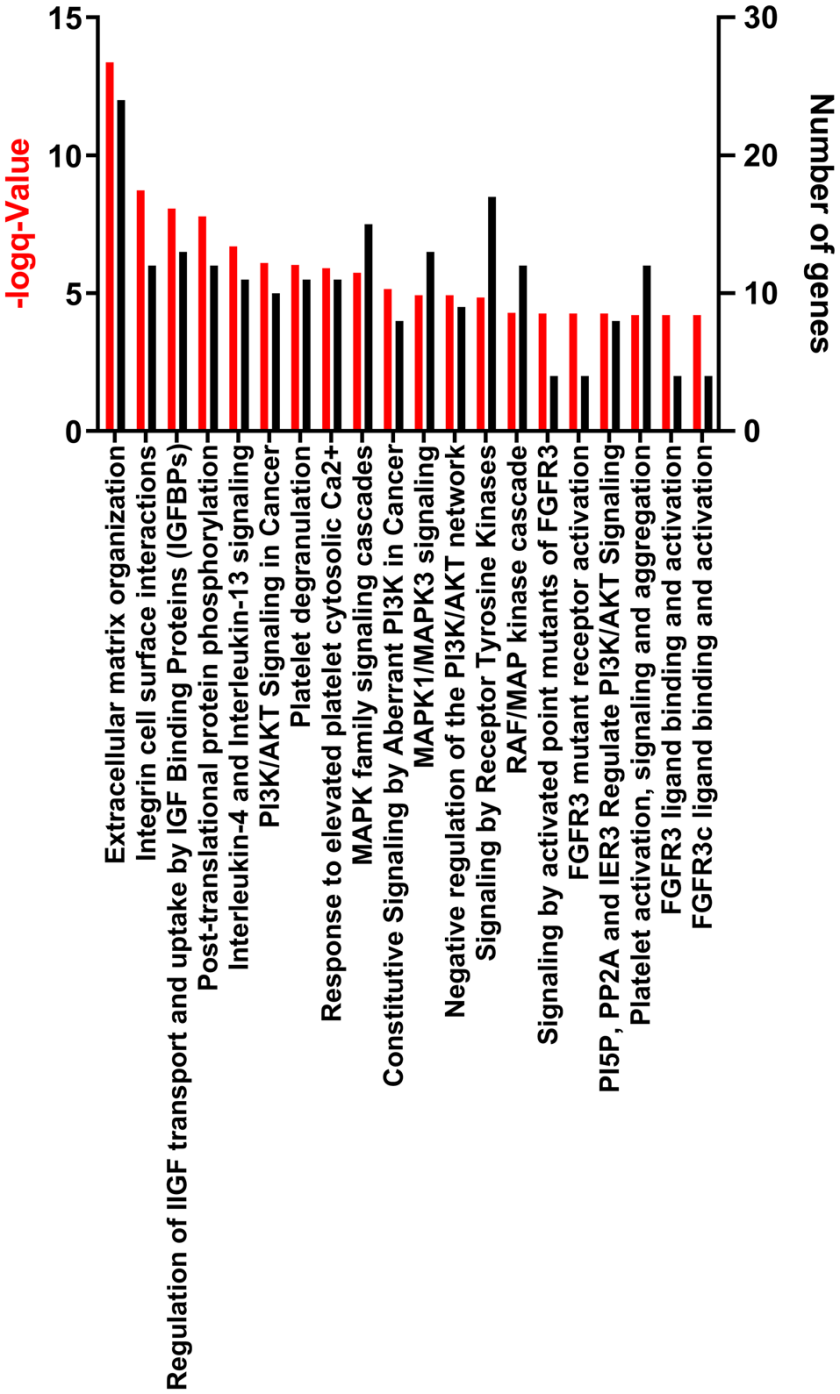
Reactome Top 20 (up) regulated pathways using pathway names and number of genes with fold-change > 2 and adjusted *p*-value < 0.01 for EXT-PNI versus Focal/Non-PNI.

Reactome pathways were created using the top 144 genes with threshold of adjusted *p*-value < 0.01 and fold-change > 2 from the comparison between EXT-PNI and Focal/Non-PNI cohorts (Fig. 5). DEGs were most significantly associated with extracellular matrix organization (R-HSA-1474244), followed by integrin cell surface interactions (R-HSA-216083), regulation of insulin-like growth factor (IGF) transport, and uptake by Insulin-like Growth Factor Binding Proteins (IGFBPs) (R-HSA-381426). Other pathways of interest include post-translational protein phosphorylation/PI3K/AKT signalling in cancer/MAPK family signalling cascade/signalling by receptor tyrosine kinases as well as platelet degranulation and platelet activation, signalling and aggregation pathways.

## Discussion

In this study we present gene expression profiling of 45 HNcSCCs with and without PNI and the initial development of a genetic signature for determining clinically significant PNI. The finding of highly stereotyped expression changes which coincide with the biological divergence of the disease suggests that, with appropriate validation, this signature could be an important tool in the diagnosis of clinically significant PNI in cSCC.

To date, the body of work examining PNI in HNcSCC using next generation sequencing technology and bioinformatics analysis is limited with the majority of work instead focusing on advanced/high-risk cSCC^1,10,11^ or metastatic disease^12,13^. An exception is the work by Warren et al.^14^ which undertook expression analysis of PNI specimens with a focus on mutations which influence p53 activation. Although this study was more limited in scope, the authors similarly observed that the genetic changes in the clinically significant PNI group were distinct to those in the focal/incidental PNI group and that there was no observable pattern of progression between the non, incidental and clinically significant PNI groups.

### Transcriptomic analysis

At a functional level, genes responsible for extracellular matrix interaction, neural development and signal transduction were observed to be overexpressed in patients with extensive PNI with a clear divide between the Focal/Non-PNI groups and the EXT-PNI group. This finding was independent of the quantity of neural tissue contained in the samples analyzed. The EXT-PNI group also demonstrated clear differences in expression across an array of genes associated with epithelial to mesenchymal transition, cellular motility, angiogenesis, cell adhesion and cellular differentiation compared to the Focal/Non-PNI group (Fig. 5).

The top 10 DEGs based on adjusted *P*-value include*, PTGIS, THBS4, SRGN, FERMT2, NR4A3, TIMP1, SAMSN1, HGF, VCAN* and *C3* (Supplementary Table 1)*.* The majority of these genes have known extracellular matrix, cell adhesion, cell motility and/or neurodevelopment interactions (Supplementary Table 4) suggesting they may play a mechanistic role in the biological process underpinning the development of extensive PNI. Studies in SCC at other sites, including mucosal SCC have demonstrated a trend towards upregulation of adhesion molecules such as *ICAM* amongst tumors with PNI as compared with those lacking PNI^15^. In Pancreatic ductal adenocarcinoma the *IGFBP* family have been described to be down-regulated in PNI as compared to those without PNI^16^. Zilberg et al.^11^ report *FGFR2* missense mutations found exclusively in primary HNcSCC with PNI (some of these specimens were included in this analysis). Other previously reported pathways or molecules with a possible mechanistic role in PNI include *BDNF, NTRK2, TWIST, NOTCH4, RET, SNAIL, NGF, NCAM1* and *CDH1*^17–19^. Of these only *NCAM1* and *CDH1,* both of which were significantly differentially expressed in the EXT-PNI versus Focal/Non-PNI comparison (Supplementary Table 1), were included in the Nanostring panel used in this study. *CDH1* encodes E-cadherin an important cell–cell adhesion molecule whose loss in epithelial tissues is associated with EMT, cell invasion and metastasis^20^. This gene was significantly downregulated in the EXT-PNI cohort and in fact was found to be one of the four uniquely downregulated genes in the EXT-PNI versus Focal-PNI comparison (refer to Fig. 1F). *NCAM1*, whose expression was significantly increased in the EXT-PNI group, encodes neural cell adhesion molecule 1. In vitro work has demonstrated that *NCAM1* is important for Schwann-cell facilitated cancer cell invasion as a potential driving mechanism for PNI and the interaction between *CDH1* downregulation and *NCAM*1 upregulation may be linked^21^. Although *CDH1* and *NCAM1* were not the top DEGs based on adjusted *P*-value, the combined changes in these DEGs should be explored further in future studies as a potential marker of risk of progression.

Interestingly, the genes uniquely up- or downregulated in EXT-PNI compared to Focal-PNI (Fig. 1F) appeared to be associated with pathways affecting extracellular matrix organization/interactions, cell adhesion and proliferation and angiogenesis but not neural development. These differential changes may give insight into the development of the pathology however further analysis with larger datasets will be required to determine the significance of this finding. The downregulated genes include those that encode either specific cell–cell adhesion molecules such as the desmosomal cadherin (*DSC2*), which has been shown to be downregulated in various malignancies correlating with increased metastasis and poorer prognosis^22^ and E-cadherin, the transcription factor ets variant 4 (*ETV4*), a regulator of keratinocyte differentiation^23^, and an epidermal growth factor (EGF) family member epiregulin (*EREG*), which is known to stimulate skin inflammation and wound healing, as well as cell proliferation and other processes contributing to cancer progression in a number of different solid tumor types^24^. Genes that were uniquely upregulated in EXT-PNI versus focal-PNI include members of the TGFB1 family (*BMP5, INHBE)* and other signalling molecules that promote cell proliferation, migration and survival *(FGF9*), a human metastasis suppressor gene (*KISS1*), and genes encoding chemokines (*CX3CL1*, *CXCL17*) and integrin subunits (*ITGAM*) which regulate leukocyte migration and play roles in angiogenesis and inflammation. Other genes included those that encode members of the proteoglycan family (*HAPLN1*, *SPOCK3*) and influence ECM structure in the tumor microenvironment and urotensin 2 (*UTS2*) which has known roles in regulation of angiogenesis^25^.

### Expression analysis in clinical practice

Expression analysis as a form of next-generation sequencing has emerged as a valuable tool in assisting treatment decisions in oncology and personalising cancer care. A recent survey revealed 75.6% of oncologists in the United States reported using next-generation sequencing to guide treatment decisions^26^. Oncotype DX^27^ and Prosigna^28^ are well known examples of clinically implemented genomic expression analysis panels which can be used to objectively inform the risk of breast cancer recurrence in lymph node negative, estrogen receptor positive tumors. As HNcSCC is treated by a large number of clinicians of varied levels of skill and experience and limited availability of specialist head and neck pathologists, the introduction of an objective panel which identifies patients who need consideration of treatment escalation is valuable, especially with regards to adjuvant radiation and systemic therapy. Further investigation of the expression analysis in the setting of pre-operative biopsies may also assist in determining operative decision making with regard to the extent of resection and inform the planning of reconstruction.

### Limitations

The main limitation of this study is the inability to test for the DEG of the identified genes in the initial specimen at first presentation of patients with extensive PNI. It is common for patients to have multiple cSCCs treated over a long history and the primary tumor of origin for extensive PNI specimens being difficult to determine. Also, due to the retrospective nature of the study it is beyond the scope of the study to determine if the gene expression changes pre-date the onset of clinically significant PNI or vice-versa. Anecdotally, FOCAL-7 and FOCAL-11 provide some hope that this may be the case. The retrospective cohort in this study does have some benefits, particularly in terms of availability of follow-up data and the ability to assemble clinical groups that are well matched based on age, sex and tumor size. Although the sample size of the groups compares favourably to previous molecular based studies of HNcSCC, the requirement to extract high quality RNA from formal-fixed paraffin embedded specimens for expression analysis does significantly impact the number cases suitable for inclusion and makes the technique technically demanding. This technical limitation will be improved through future prospective studies where RNA quality can be controlled for through protocolised tissue sampling and modern specimen processing.

From a transcriptome perspective, the relatively small number of neurotropism specific genes included in the PanCancer progression panel limits the ability for this study to provide more definitive guidance regarding neurotropism. For instance, other than *NCAM1*, certain well-known neurotropic genes such as those encoding neurotropin tyrosine kinase 1, 2 and 3 (*NTRK1, NTRK2, NTRK3, *respectively), are not included, precluding a comprehensive analysis of factors influencing neurotropism.

Future prospective validation of our findings in a larger cohort is essential prior to clinical implementation. In particular, future studies which apply this panel to paired biopsy and resection specimens will be critical to determining whether there is a role for this tool in pre-operative assessment to enable individualised treatment regarding extent of resection and/or the addition of neoadjuvant treatment.

### Conclusions

Nanostring PanCancer analysis of HNcSCC demonstrates significantly different gene expression profiles in HNcSCC with extensive PNI likely to develop recurrence as compared with patients without PNI or those with only focal PNI. PNS tracking along large nerves with clinical manifestations is a clinically and molecularly distinct disease as compared with histologically identified, clinically asymptomatic PNI. In fact, while the cohort is relatively small, there are no differences in the gene expression profile of HNcSCC with histologically identified focal PNI and HNcSCC without PNI. The findings of our study would have significant clinical implications if differential gene expression could be identified at the time of the initial biopsy so that there can be appropriate escalation of treatment as well as surveillance.

## Methods

Following institutional Human Research Ethics committee approval (Royal Prince Alfred Research Ethics and Governance Office, Australia; University of Wollongong Health and Medical Human Research Ethics Committee, Wollongong NSW, Australia, (UOW/ISLHD HREC 14/397), patients with HNcSCC treated with curative intent between 2008 and 2018 were identified from the prospectively collected database held at the Sydney Head and Neck Cancer Institute. All participants provided informed consent as part of established tissue banking and research protocols covering this project and the study was conducted in accordance with all appropriate guidelines and regulations. Cases were included based on volume and quality of RNA meeting adequate requirements for reliable expression analysis. Cases that did not show histopathologic evidence of PNI in the entire examined resection specimen of the primary HNcSCC were classified as Non-PNI. These cases also required a minimum follow up of 4 years for inclusion in the study. Cases that showed histologic evidence of a single focus of PNI involving a nerve twig not greater than 0.2 mm in maximum diameter in the entire examined resection specimen of the primary HNcSCC were classified as Focal-PNI. Cases that showed histologic evidence of multiple foci of PNI or involvement of a named nerve were classified as extensive (EXT-PNI). In total, 45 cases of HNcSCC met the selection criteria (Table 1).

**Figure 6:**
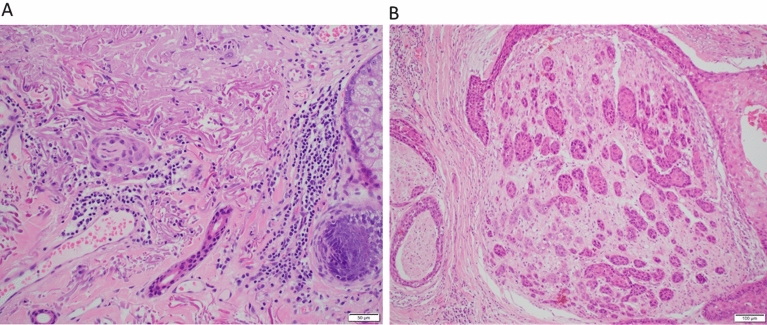
Representative images of H&E stained specimens with perineural invasion. Left image demonstrates a single focus of perineural invasion within the dermis beyond the invasive front of the tumour (×20). Right image demonstrates extensive perineural invasion of a major nerve (×10).

The histopathology slides and paraffin blocks were retrieved from the archives of the Department of Tissue Pathology and Diagnostic Oncology at Royal Prince Alfred Hospital, Sydney, New South Wales, Australia. A complete histopathology review was performed and the tumor size, depth of invasion, lympho-vascular and PNI, nerve content in specimens, bone involvement and margins of resection were recorded. Highly cellular areas of the tumor with a neoplastic cell content of 30–90% and without necrosis, keratin, inflammatory infiltrate or hemorrhage were identified. Examples of these specimens are shown in Fig. 6.

Clinical follow up was obtained from the Sydney Head and Neck Cancer Institute database and clinical records for each case. Categorical data was compared using the χ^2^-test and continuous data was analysed the student’s t-test. A *p*-value of < 0.05 was determined as significant.

### RNA isolation and quality assessment

Tissue selected as described above was macro-dissected from the blocks for RNA extraction. using the AllPrep DNA/RNA FFPE Kit (Qiagen, Venlo, Netherlands). All specimens were quantified using the NanoDrop (ND1000, ThermoFisher Scientific, Waltham, MA, USA). RNA specimens with A260/280 ratios between 1.8 and 2.1 were then analysed prior to NanoString preparation work-flow to determine RNA quality with a Qubit 3.0 RNA Hi-Sensitivity analysis kit (Life Technologies, Carlsbad, CA, USA).

### Gene expression assays

A total of 150 ng of purified RNA was run on the nCounter Sprint system and the commercially available PanCancer Progression panel CodeSets (NanoString Technologies, Seattle, WA, USA), which contains 740 target genes and 30 “housekeeping” genes (https://www.nanostring.com) as per the manufacturer's instructions. Data was preprocessed with nSolver Analysis Software 4.0 (NanoString) following all recommended quality control steps. This included background correction by spiked-in negative control probes and data normalization using positive control normalization probes and CodeSet Content Normalization, which uses housekeeping genes to apply a sample-specific correction factor to the target probes within a sample lane. Calibrator samples were incorporated to correct lot to lot variation in CodeSets.

### Gene expression data analysis and visualization

nSolver normalized data was used for all statistical analysis. Limma package^29^ was used to identify the differentially expressed genes (DEGs) between the PNI groups. eBayes function was used to compute moderated t-statistics, moderated F-statistic and log-odds of differential expression, which is by empirical Bayes moderation of the standard errors towards a common value. The top DEGs were selected based on both fold change between the compared 2 groups and the *p*-values adjusted for multiple testing with Benjamini–Hochberg method. They were then used for clustering analysis with the gene profile patterns visualized through heatmaps. For all the clustering analyses presented in this paper, Euclidean distance was used to measure the dissimilarity between each pair of the observations. See Supplementary Table 1 for specimen identifiers.

To identify pathway associations across REACTOME pathway lists (http://www.reactome.org/download/current/, source dated 2020-03-11) ReactomePA^30^ was used with the DEGs from the comparison between EXT-PNI and Focal/Non-PNI cohorts for gene enrichment analysis. A hypergeometric test was used to determine whether the list of genes associated with REACTOME pathway is larger than expected by chance.

### Sensitivity and specificity analysis

A score to classify HNcSCC cohorts for EXT-PNI was developed based on the top ten most significant DEGs based on adjusted *p*-values. Gene specific thresholds were calculated using the 95th centile for the Non- and Focal-PNI values. This threshold was then applied to each sample with a score of 1 being attributed to values greater than the 95th centile and a score of 0 being applied to values less than then 95th centile. This approach was used, as opposed to a standardised numerical fold change cut-off, because some genes demonstrate highly significant upregulation or downregulation with lower absolute fold change numbers. A sensitivity and specificity analysis was calculated based on the sum of score values. Samples with total score values ≥ 2 were defined as high risk for clinically significant progression of PNI, while samples with total score values ≤ 1 were defined as low risk.

## Supplementary Information

Supplementary Dataset 1.

Supplementary Information.
