# Supplementary material for: Gene expression profiling of perineural invasion in head and neck cutaneous squamous cell carcinoma
Source: Sci Rep. 2021 Jun 23;11:13192. doi: 10.1038/s41598-021-92335-4 (PMC8222302; doi:10.1038/s41598-021-92335-4)
Supplement: Supplementary file 2 — Supplementary Information. [file 41598_2021_92335_MOESM2_ESM.pdf]

Supplementary Tables and Figures

Manuscript Title:

GENE EXPRESSION PROFILING OF PERINEURAL INVASION IN HEAD AND NECK CUTANEOUS SQUAMOUS CELL CARCINOMA.

Authors:

Timothy J Eviston, Elahe Minaei, Simon A Mueller, Navid Ahmadi, Bruce Ashford, Jonathan R Clark, Nicholas West, Ping Zhang, Ruta Gupta, Marie Ranson

Supplementary Table 1. List of 144 significantly DEG between extensive (EXT) PNI versus combined focal and non PNI (FOC_NON) based on adjusted P-values <0.01, ranked by increasing *P*-values. Grey highlighted genes are downregulated in EXT PNI with fold change < -2. Bolded genes are DEG with fold change >3 or < -3 (n = 70). LogFC = log2 fold change. CI.L/R = confidence intervals left/right.

	HUGO	Average_EXT	Average_FOC_NON	logFC	CI.L	CI.R	AveExpr	t	P.Value	adj.P.Val	B
1	***PTGIS***	8.176542	3.911408	4.265134	3.057936	5.472331	6.280926	7.109034	5.81E-09	4.3E-06	10.41069
2	***THBS4***	9.00886	4.012144	4.996716	3.534998	6.458434	6.788097	6.878239	1.3E-08	4.79E-06	9.646227
3	***SRGN***	12.18703	10.15644	2.030587	1.403969	2.657205	11.28455	6.520409	4.51E-08	1.11E-05	8.458462
4	***FERMT2***	8.993999	7.276204	1.717795	1.150489	2.285101	8.230535	6.092708	2E-07	2.99E-05	7.039322
5	***NR4A3***	8.541688	5.711376	2.830313	1.895107	3.765518	7.283772	6.089529	2.02E-07	2.99E-05	7.028792
6	***TIMP1***	11.85543	9.6565	2.198932	1.45043	2.947434	10.87813	5.911193	3.76E-07	4.63E-05	6.438852
7	*SAMSN1*	8.602073	7.071685	1.530389	1.002459	2.058318	7.921901	5.832864	4.93E-07	5.21E-05	6.180302
8	***HGF***	6.548982	3.692766	2.856216	1.836671	3.875761	5.279552	5.636905	9.7E-07	8.97E-05	5.535427
9	***VCAN***	10.04003	8.191016	1.849012	1.172124	2.5259	9.218245	5.496411	1.57E-06	0.000122	5.075177
10	***C3***	10.04841	6.81079	3.237621	2.049321	4.425922	8.609468	5.482214	1.65E-06	0.000122	5.028781
11	***IGFBP7***	13.07847	11.3441	1.734371	1.094327	2.374415	12.30764	5.45241	1.83E-06	0.000123	4.931453
12	***AKAP12***	8.538593	5.737349	2.801244	1.733657	3.868831	7.293595	5.279635	3.31E-06	0.000204	4.36935
13	*MS4A4A*	9.520656	8.025336	1.49532	0.921147	2.069492	8.85607	5.240199	3.78E-06	0.000207	4.241595
14	*LHFP*	8.942235	7.475332	1.466903	0.902555	2.031252	8.290278	5.230104	3.91E-06	0.000207	4.208929
15	***CDH2***	5.481306	2.811874	2.669432	1.63051	3.708353	4.294892	5.170022	4.8E-06	0.000224	4.014806
16	***SLIT2***	6.829539	4.184884	2.644655	1.614805	3.674506	5.654137	5.167152	4.85E-06	0.000224	4.005547
17	***FAP***	9.81836	7.676393	2.141967	1.300917	2.983016	8.866375	5.124452	5.61E-06	0.000244	3.867939
18	*ITGA5*	10.2619	9.106893	1.155003	0.698652	1.611353	9.748561	5.092611	6.24E-06	0.00025	3.765509
19	***CDH11***	10.7942	9.018946	1.775257	1.072582	2.477933	10.0052	5.083499	6.44E-06	0.00025	3.736229
20	*HLA.DPB1*	12.20502	10.81758	1.387442	0.836685	1.938198	11.58838	5.068873	6.77E-06	0.00025	3.689256
21	***FGFR3***	8.094096	10.70626	-2.61217	-3.65298	-1.57135	9.255059	-5.04989	7.21E-06	0.000254	3.628351
22	*SERPING1*	11.61714	10.11045	1.506696	0.902587	2.110805	10.9475	5.018415	8.02E-06	0.00027	3.527479
23	***APOD***	10.46412	6.511564	3.952558	2.353878	5.551238	8.70743	4.97477	9.3E-06	0.00029	3.387904
24	***FGF18***	4.890604	2.313239	2.577365	1.534264	3.620466	3.745109	4.971712	9.39E-06	0.00029	3.378137
25	*PLXND1*	9.777288	8.725009	1.052279	0.624724	1.479834	9.309608	4.952166	1E-05	0.000297	3.315751

26	***MPDZ***	8.066133	6.332897	1.733236	1.023495	2.442977	7.295806	4.91376	1.14E-05	0.000325	3.193372
27	*JAM3*	8.947986	7.407351	1.540635	0.90642	2.17485	8.26326	4.887868	1.25E-05	0.000333	3.111027
28	***ZFPM2***	4.633681	2.242943	2.390738	1.405794	3.375683	3.571131	4.88401	1.26E-05	0.000333	3.098769
29	***SULF1***	10.64963	8.627711	2.021922	1.18476	2.859083	9.751001	4.859721	1.37E-05	0.000349	3.021657
30	***DDR2***	8.769871	6.950236	1.819636	1.06441	2.574862	7.961144	4.848013	1.42E-05	0.000351	2.984529
31	***SFRP2***	11.90511	9.631576	2.273538	1.326233	3.220843	10.89465	4.829126	1.52E-05	0.000362	2.924691
32	*SERPINF1*	11.65157	10.09272	1.55885	0.900421	2.217279	10.95875	4.76378	1.89E-05	0.000436	2.718227
33	***TBXA2R***	4.736541	2.327017	2.409525	1.387441	3.431608	3.665642	4.743525	2.02E-05	0.000452	2.654413
34	***MGP***	10.93773	8.228106	2.709628	1.551047	3.86821	9.733455	4.705865	2.29E-05	0.000497	2.536001
35	***CYP1B1***	7.29957	4.774274	2.525296	1.434826	3.615765	6.177216	4.659667	2.66E-05	0.000563	2.391176
36	*LY96*	8.985937	7.821156	1.164781	0.657489	1.672073	8.468257	4.620008	3.04E-05	0.000624	2.267237
37	*FGFR1*	9.116971	7.730922	1.38605	0.778541	1.993558	8.500949	4.59074	3.34E-05	0.000669	2.17601
38	*FGL2*	8.677779	7.238421	1.439358	0.804511	2.074206	8.038064	4.562007	3.68E-05	0.000713	2.086652
39	***FN1***	13.04137	11.01434	2.027021	1.130222	2.923819	12.14047	4.54799	3.85E-05	0.000713	2.043132
40	*ICAM1*	9.93147	8.502919	1.42855	0.796501	2.0606	9.296559	4.547792	3.85E-05	0.000713	2.042515
41	***NR4A1***	9.925446	7.351176	2.57427	1.428029	3.720512	8.781326	4.518917	4.24E-05	0.000764	1.953023
42	***NDNF***	5.978693	3.15764	2.821053	1.557249	4.084856	4.724891	4.491464	4.63E-05	0.000817	1.868131
43	*PLXNC1*	9.103322	7.994631	1.108691	0.610895	1.606487	8.610571	4.48142	4.79E-05	0.000824	1.837122
44	***SPARCL1***	10.72327	8.756495	1.966778	1.080653	2.852904	9.84915	4.465976	5.04E-05	0.000847	1.78949
45	***BNC2***	5.981212	3.900942	2.080271	1.136124	3.024417	5.056647	4.433399	5.6E-05	0.000921	1.689225
46	***FOXC2***	6.089285	3.786533	2.302752	1.25531	3.350195	5.065839	4.423574	5.78E-05	0.000931	1.65904
47	***PTX3***	5.655791	2.897084	2.758707	1.500468	4.016947	4.429699	4.411625	6.01E-05	0.000947	1.622365
48	*ITGB2*	9.390146	8.12456	1.265586	0.682801	1.84837	8.827663	4.369582	6.89E-05	0.001043	1.493638
49	***FBLN1***	11.6077	9.763386	1.844315	0.994191	2.694439	10.788	4.365249	6.99E-05	0.001043	1.480399
50	*OLFML2B*	9.640894	8.409339	1.231555	0.663367	1.799743	9.093536	4.361322	7.08E-05	0.001043	1.468406
51	*PECAM1*	9.79908	8.52566	1.27342	0.685301	1.861539	9.233115	4.35675	7.19E-05	0.001043	1.454447
52	*AKAP2*	9.879174	8.64341	1.235764	0.663769	1.807758	9.329946	4.347104	7.42E-05	0.001055	1.425015
53	*VEZF1*	8.660196	7.79851	0.861687	0.460969	1.262404	8.277224	4.326805	7.92E-05	0.001106	1.363167
54	*PXDN*	9.676528	8.470313	1.206214	0.644464	1.767964	9.140432	4.320536	8.08E-05	0.001107	1.344091

55	***OGN***	8.456998	5.104503	3.352495	1.777635	4.927354	6.967	4.28334	9.11E-05	0.001226	1.231138
56	*WIPF1*	9.732064	8.7522	0.979864	0.51705	1.442678	9.296569	4.260058	9.82E-05	0.001276	1.160641
57	***SLC2A1***	8.811934	10.97299	-2.16105	-3.18183	-1.14028	9.772403	-4.25981	9.83E-05	0.001276	1.15989
58	*ZCCHC24*	8.871509	7.596307	1.275202	0.669988	1.880416	8.304752	4.239615	0.000105	0.001337	1.098876
59	*CDS1*	7.299836	8.845294	-1.54546	-2.27983	-0.81108	7.986706	-4.23443	0.000107	0.001337	1.08324
60	***CAMK2A***	4.153675	1.952525	2.20115	1.152569	3.24973	3.175386	4.223807	0.00011	0.001361	1.051203
61	*ENPP2*	8.705625	7.225327	1.480298	0.772597	2.187999	8.047715	4.208774	0.000116	0.001405	1.005936
62	*C3AR1*	8.185371	7.042688	1.142684	0.593793	1.691574	7.677512	4.188868	0.000123	0.001464	0.946101
63	***ABI3BP***	9.911305	7.843304	2.068002	1.073863	3.062141	8.992194	4.185623	0.000125	0.001464	0.936361
64	*LUM*	13.37394	12.04099	1.332957	0.689899	1.976016	12.78152	4.170825	0.000131	0.001501	0.891974
65	*DCN*	12.50047	11.01845	1.482021	0.766402	2.19764	11.84179	4.167048	0.000132	0.001501	0.880657
66	*FLI1*	8.568956	7.144349	1.424607	0.736083	2.113131	7.935797	4.163247	0.000134	0.001501	0.869272
67	***VCAM1***	8.581596	6.850553	1.731043	0.88403	2.578056	7.812244	4.112198	0.000158	0.00174	0.716817
68	*SERPINA1*	8.363761	6.955238	1.408524	0.715046	2.102001	7.737751	4.086843	0.000171	0.001858	0.641408
69	*BAG2*	8.427783	7.148475	1.279308	0.646336	1.912281	7.859202	4.066742	0.000182	0.001929	0.581776
70	*ZFYVE16*	8.548574	7.77575	0.772823	0.390357	1.15529	8.205097	4.065773	0.000182	0.001929	0.578905
71	***FHL1***	9.364727	7.603661	1.761065	0.884877	2.637254	8.582031	4.044214	0.000195	0.002036	0.515104
72	*NR3C1*	10.41566	9.622065	0.793593	0.397189	1.189997	10.06295	4.028246	0.000205	0.002052	0.467952
73	***MS4A6A***	6.772603	4.597062	2.175541	1.088538	3.262543	5.805696	4.027105	0.000206	0.002052	0.464586
74	***CXADR***	8.695033	10.29111	-1.59608	-2.39389	-0.79827	9.4044	-4.02542	0.000207	0.002052	0.459631
75	*EVI2A*	6.894859	5.785167	1.109692	0.554678	1.664706	6.401663	4.023046	0.000209	0.002052	0.452615
76	***CX3CL1***	7.403502	5.389046	2.014457	1.006175	3.022738	6.508188	4.020058	0.000211	0.002052	0.443809
77	***DSC2***	10.22294	11.79555	-1.57261	-2.36617	-0.77905	10.92188	-3.98747	0.000234	0.002244	0.347955
78	***MEOX2***	7.06517	5.010674	2.054496	0.999201	3.10979	6.15206	3.917308	0.000291	0.002759	0.142807
79	***UTS2***	3.977076	2.059202	1.917873	0.930207	2.90554	3.124688	3.907202	0.0003	0.002811	0.113406
80	***TBX1***	6.853872	8.970473	-2.1166	-3.21311	-1.02009	7.794584	-3.88402	0.000323	0.002983	0.046092
81	*THBS1*	11.45082	9.904541	1.546277	0.740957	2.351597	10.76358	3.863446	0.000344	0.003136	-0.01346
82	*MMP2*	11.75157	10.48994	1.261629	0.603549	1.919709	11.19085	3.857527	0.00035	0.003136	-0.03057
83	*SRPX2*	7.393977	5.871319	1.522658	0.727562	2.317754	6.71724	3.853355	0.000355	0.003136	-0.04262

84	***GJA5***	5.449455	3.84465	1.604806	0.766519	2.443093	4.736208	3.851999	0.000356	0.003136	-0.04653
85	***CLU***	10.58483	8.542835	2.041998	0.974311	3.109685	9.677278	3.848288	0.00036	0.003136	-0.05724
86	***ADAMTS8***	3.11158	1.296359	1.81522	0.862186	2.768255	2.304815	3.832454	0.000378	0.003255	-0.10289
87	***PRSS8***	6.037842	8.35102	-2.31318	-3.52937	-1.09699	7.065921	-3.82704	0.000385	0.003272	-0.11846
88	***ASPN***	9.327385	6.732426	2.59496	1.227027	3.962892	8.17407	3.817001	0.000397	0.003337	-0.14734
89	*DENND5A*	8.312798	7.607362	0.705437	0.333016	1.077857	7.999271	3.811362	0.000404	0.003357	-0.16354
90	***NRXN3***	3.713998	1.723964	1.990035	0.93786	3.042209	2.829538	3.805652	0.000411	0.003378	-0.17993
91	***TFPI2***	6.876605	3.7028	3.173804	1.492142	4.855467	5.466025	3.797498	0.000421	0.003426	-0.20332
92	*C1S*	10.46129	9.201829	1.259463	0.591441	1.927484	9.90153	3.793594	0.000426	0.00343	-0.2145
93	*ITGAM*	6.869665	5.309823	1.559842	0.730339	2.389345	6.176402	3.783719	0.00044	0.003497	-0.24277
94	***TPM2***	12.82356	11.23126	1.592298	0.744544	2.440053	12.11587	3.779294	0.000446	0.003508	-0.25543
95	*SORD*	8.785962	9.743997	-0.95803	-1.47049	-0.44557	9.211755	-3.76164	0.00047	0.003664	-0.30583
96	*QKI*	11.17532	10.53903	0.636283	0.295311	0.977255	10.89252	3.754804	0.00048	0.003702	-0.32533
97	*PMP22*	9.854247	8.911144	0.943103	0.436876	1.449329	9.43509	3.748611	0.000489	0.003734	-0.34297
98	*CRISPLD2*	9.970885	8.908296	1.06259	0.490989	1.63419	9.498623	3.740496	0.000502	0.003789	-0.36606
99	*CCDC80*	11.99446	10.48601	1.508448	0.693302	2.323594	11.32404	3.723499	0.000528	0.00395	-0.41434
100	*HIPK1*	9.483144	8.865434	0.61771	0.282627	0.952793	9.208606	3.709268	0.000552	0.004084	-0.45468
101	*GSN*	11.77425	10.71099	1.063264	0.485773	1.640756	11.30169	3.70469	0.00056	0.0041	-0.46763
102	*PLEKHO1*	10.80888	9.918621	0.890255	0.404339	1.376172	10.41321	3.686457	0.000592	0.004291	-0.51916
103	***FGF2***	5.314698	3.389958	1.92474	0.872609	2.976871	4.459258	3.680938	0.000602	0.004321	-0.53472
104	*CTNNB1*	11.66443	10.90388	0.760555	0.340369	1.180741	11.32641	3.642045	0.000677	0.004815	-0.64408
105	*CTSL*	9.062662	7.926084	1.136578	0.504892	1.768265	8.557516	3.620378	0.000722	0.005062	-0.70473
106	*WWTR1*	10.09973	9.094521	1.005204	0.44634	1.564069	9.652968	3.619129	0.000725	0.005062	-0.70822
107	***EREG***	3.992915	6.156164	-2.16325	-3.37265	-0.95385	4.954359	-3.5991	0.00077	0.005325	-0.76411
108	***VSIG4***	9.150768	7.220556	1.930212	0.850181	3.010244	8.292896	3.596043	0.000777	0.005325	-0.77261
109	*NRP2*	10.29514	9.162454	1.132687	0.496301	1.769072	9.791724	3.581341	0.000812	0.00551	-0.8135
110	*VIM*	14.3035	13.24121	1.062294	0.464993	1.659595	13.83137	3.578554	0.000819	0.00551	-0.82124
111	*NFATC2*	9.350565	8.003056	1.347509	0.588515	2.106503	8.751672	3.572314	0.000834	0.005563	-0.83856
112	*PTK6*	6.397552	7.772476	-1.37492	-2.15078	-0.59906	7.008629	-3.56575	0.000851	0.005601	-0.85675

113	***SCG2***	4.170552	2.264377	1.906175	0.830019	2.982331	3.323364	3.56405	0.000855	0.005601	-0.86147
114	*ITGA1*	9.230424	8.299875	0.930548	0.404452	1.456644	8.816847	3.559018	0.000868	0.005631	-0.8754
115	*PTPRC*	7.539817	6.343455	1.196362	0.519494	1.873231	7.008101	3.556432	0.000875	0.005631	-0.88256
116	***BMP5***	4.338512	2.272708	2.065805	0.894832	3.236777	3.420377	3.549759	0.000893	0.005694	-0.90102
117	*KCNJ8*	7.259001	5.957911	1.30109	0.56116	2.04102	6.680739	3.538125	0.000924	0.005845	-0.93315
118	*FLT1*	7.917472	6.390603	1.526869	0.653125	2.400613	7.238863	3.516202	0.000986	0.006157	-0.99353
119	*PDGFRB*	10.36	9.206587	1.153418	0.493142	1.813694	9.847374	3.514936	0.00099	0.006157	-0.99701
120	***PTPRB***	4.070078	2.092443	1.977635	0.841933	3.113337	3.191129	3.50379	0.001023	0.006305	-1.02762
121	***FGF9***	3.854895	2.206798	1.648098	0.700982	2.595213	3.122407	3.501357	0.001031	0.006305	-1.0343
122	***IL6***	5.312837	2.478713	2.834124	1.196531	4.471717	4.053226	3.482323	0.001091	0.006616	-1.08642
123	***NCAM1***	5.286764	2.973735	2.313029	0.973443	3.652616	4.258752	3.474294	0.001117	0.006671	-1.10836
124	*AKT3*	9.34515	8.234738	1.110412	0.467264	1.753561	8.851634	3.473997	0.001118	0.006671	-1.10917
125	*PKN1*	9.4174	8.467567	0.949833	0.398245	1.501421	8.995252	3.464885	0.001148	0.006765	-1.13403
126	*ITGB3*	7.432487	5.993123	1.439364	0.603248	2.275479	6.79277	3.46386	0.001152	0.006765	-1.13683
127	***ETV4***	4.855539	6.580971	-1.72543	-2.72977	-0.7211	5.622398	-3.45681	0.001176	0.006852	-1.15605
128	***CNN1***	4.006901	1.738547	2.268354	0.943728	3.59298	2.998744	3.44567	0.001215	0.007026	-1.18634
129	*KIAA1462*	8.18355	7.222046	0.961504	0.398867	1.524141	7.756215	3.438578	0.001241	0.007078	-1.2056
130	***S100A7***	7.829611	11.93479	-4.10518	-6.50788	-1.70247	9.654133	-3.43786	0.001243	0.007078	-1.20756
131	***CHRDL1***	5.909359	2.905235	3.004124	1.242924	4.765324	4.574192	3.432143	0.001265	0.007143	-1.22305
132	*CHRNA7*	5.154154	3.700685	1.453469	0.598223	2.308715	4.508168	3.419565	0.001312	0.007287	-1.25712
133	*CDK14*	8.975443	7.816931	1.158513	0.475963	1.841062	8.460549	3.415254	0.001329	0.007287	-1.26878
134	*NAA15*	7.492039	8.442593	-0.95055	-1.51071	-0.3904	7.914508	-3.41448	0.001332	0.007287	-1.27088
135	*ESRP1*	9.659245	10.81963	-1.16039	-1.84446	-0.47631	10.17497	-3.41314	0.001337	0.007287	-1.2745
136	*MAP2K4*	7.51672	8.574664	-1.05794	-1.68173	-0.43416	7.986917	-3.41257	0.001339	0.007287	-1.27602
137	***PLA2G2A***	8.598091	5.321259	3.276832	1.328358	5.225307	7.141721	3.383886	0.001456	0.007867	-1.35336
138	*CDH1*	8.835033	10.33455	-1.49952	-2.39273	-0.60631	9.501487	-3.37796	0.001482	0.007925	-1.36927
139	***POSTN***	11.85721	10.20854	1.64867	0.666155	2.631184	11.12447	3.376378	0.001489	0.007925	-1.37353
140	*ALB*	2.794902	1.350269	1.444633	0.57735	2.311915	2.152843	3.351608	0.0016	0.008457	-1.4399
141	*JUN*	10.67711	9.560218	1.11689	0.439111	1.794669	10.18071	3.315724	0.001775	0.009317	-1.53553

142	***ACTG2***	6.674539	3.992938	2.681601	1.046505	4.316697	5.482716	3.299949	0.001858	0.009682	-1.57737
143	*GPR124*	6.577779	5.192164	1.385616	0.537935	2.233296	5.96195	3.289022	0.001917	0.009867	-1.60628
144	*CEP170*	9.061795	8.507533	0.554262	0.215128	0.893396	8.815457	3.288517	0.00192	0.009867	-1.60762

Supplementary Table 2. PNI cohort categories and nerve proportions. Green highlighted EXT specimens grouped with major focal/non-PNI cohort using fold change ± 2 stratification (refer to Figure 3).

PNI ID	% Nerve proportion*	Age, sex, primary site and additional comments
NONPNI-1	-	63yo male, scalp. Large nodal mets. CLL
NONPNI-2	-	66yo male. Scalp. Large nodal mets.
NONPNI-3	-	83yo male. Scalp. Marked inflammatory changes and desmoplasia.
NONPNI-4	-	76yo male. Ear.
NONPNI-5	-	73yo male. Preauricular.
NONPNI-6	-	73yo male. Ear
NONPNI-7	-	74yo male. Ear.
NONPNI-8	-	53yo female. Scalp.
NONPNI-9	-	59yo male. Preauricular.
FOCAL-1	1%	74yo male. Ear.
FOCAL-2	0%	92yo female. Forehead/scalp.
FOCAL-3	0.50%	65yo male. Upper lip.
FOCAL-4	0.5-1%	62yo male. Postauricular. Nodal mets.
FOCAL-5	0.5-1%	77yo male. Ear. Nodal mets.
FOCAL-6	<1%	58yo male. Neck.
FOCAL-7	<1%	65yo male. Cheek. Nodal mets.
FOCAL-8	<1%	47yo male. Cheek/parotid.
FOCAL-9	<1%	38yo male. Lip.
FOCAL-10	1%	83yo male. Preauricular.
FOCAL-11	<1%	62yo male. Temporoparietal.
EXT-1	20-30%	66 female. Nose.
EXT-2	30%	73yo female. Cheek.
EXT-3	50-60%	43 male. Preauricular.

EXT-4	20-30%	63yo male. Forehead.
EXT-5	40-50%	67yo male. Cheek.
EXT-6	1%	69yo male. Ear.
EXT-7	5%	71yo male. Cheek.
EXT-8	1%	74yo male. Forehead.
EXT-9	1-5%	82yo male. Scalp.
EXT-10	1-5%	84yo male. Postauricular.
EXT-11	1%	88yo male. Forehead.
EXT-12	1%	54yo male. Scalp.
EXT-13	1-5%	89 female. Forehead.
EXT-14	1-5%	72yo male. Ear.
EXT-15	1%	82yo male. Scalp.
EXT-16	1%	59yo male. Temporal.
EXT-17	80%	63yo male. Cheek.
EXT-18	1%	84yo male. Parotid/Cheek.
EXT-19	1%	38yo male. Lip.
EXT20	30 - 40%	68yo male. Cheek.
EXT21	1 - 2%	88yo male. Forehead.
EXT22	2 -5%	55yo male. Preauricular.
EXT23	1%	78yo male. Nose.
EXT24	20 - 30%	86yo female. Preauricular.
EXT25	60 - 70%	58yo male. Cheek.

* Nerve proportion defined as the percentage of nerve tissue contained in the cored slide undergoing expression analysis.

Supplementary Table 3. Differential gene expression in EXT-PNI cohorts based on nerve proportion in the specimens showing each genes log2 fold change >1 or < -1 of extensive PNI with high (>10%, n = 9) versus low (<10%, n = 16) nerve involvement.

	HUGO	Log2 fold change	std error (log2)	Lower confidence limit (log2)	Upper confidence limit (log2)	Linear fold change	Lower confidence limit (linear)	Upper confidence limit (linear)	P-value	adj.P. Val
1	*TMPRSS4*	-4.11	0.879	-5.83	-2.39	0.0579	0.0176	0.191	0.000105	0.504
2	*PRSS22*	-2.72	0.663	-4.02	-1.42	0.152	0.0616	0.374	0.000437	0.889
3	*ITGB8*	1.71	0.428	0.875	2.55	3.28	1.83	5.86	0.000554	0.889
4	*EVPL*	-2.13	0.578	-3.27	-0.999	0.228	0.104	0.5	0.00121	1
5	*KRT14*	-1.84	0.524	-2.86	-0.809	0.28	0.137	0.571	0.00192	1
6	*NOTCH1*	-0.951	0.277	-1.49	-0.408	0.517	0.355	0.754	0.00228	1
7	*CLDN7*	-2.36	0.711	-3.76	-0.967	0.195	0.074	0.512	0.00299	1
8	*IL1RN*	-1.43	0.433	-2.27	-0.577	0.372	0.207	0.67	0.00318	1
9	*FGFR3*	-2.07	0.684	-3.41	-0.727	0.239	0.0943	0.604	0.00606	1
10	*DPT*	-2.26	0.76	-3.75	-0.774	0.208	0.0742	0.585	0.00671	1
11	*RAB25*	-2.12	0.718	-3.53	-0.714	0.23	0.0868	0.61	0.00711	1
12	*DSC2*	-1.48	0.506	-2.48	-0.493	0.357	0.18	0.71	0.00742	1
13	*CXADR*	-1.5	0.525	-2.53	-0.473	0.353	0.173	0.72	0.00884	1
14	*ITGA11*	-2.19	0.768	-3.69	-0.681	0.22	0.0774	0.624	0.00913	1
15	*MEG3*	-1.2	0.423	-2.03	-0.373	0.435	0.245	0.772	0.00924	1
16	*IGFBP4*	1.66	0.606	0.474	2.85	3.16	1.39	7.21	0.0116	1
17	*ALDOA*	-0.861	0.314	-1.48	-0.245	0.551	0.359	0.844	0.0116	1
18	*VWA1*	1.72	0.632	0.484	2.96	3.3	1.4	7.79	0.0121	1
19	*TWIST2*	-1.19	0.443	-2.06	-0.324	0.438	0.24	0.799	0.013	1
20	*CDK14*	1.11	0.412	0.299	1.91	2.15	1.23	3.77	0.0132	1

Supplementary Table 4. Top ten genes according to adjusted *P*-values with known protein, gene function and disease associations.

Gene Symbol[1]	Protein	Function[2]	Associations[3]	Neural development	Extracellular matrix
PTGIS	prostaglandin I2 synthetase	Prostacyclin production. Prostacyclin is a vasodilator and inhibitor of platelet aggregation.	Hypertension. Pulmonary hypertension. Alzheimer's disease.	+	
THBS4	thrombospondin 4	Extracellular calcium binding. Cell proliferation, adhesion and migration.	Prostate cancer. Nephroma.	+	+
SRGN	serglycin	Platelet secretory granule proteoglycan. Inflammation and apoptosis.	Mucinoses.		
FERMT2	fermitin family member 2	Cell-ECM adhesion molecule which facilitates cell shape modulation	Kindler syndrome (epidermolysis bullosa). Leukocyte Adhesion Deficiency.	+	+
NR4A3	nuclear receptor subfamily 4 group A member 3	Neural development and cAMP signalling.	Chondrosarcoma.	+	
TIMP1	TIMP metallopeptidase inhibitor 1	Matrix metalloproteinase (MM) inhibitor. Involved in ECM degradation and stromal cell apoptosis. Growth factor.	Lung giant cell carcinoma. Oral submucous fibrosis.		+

SAMSN1	SAM domain, SH3 domain and nuclear localization signals 1	Cytoplasmic protein involved in signalling, cell spreading and cell polarization.	Negative regulator of B cell activation.		
HGF	hepatocyte growth factor	Regulates cell growth, motility and morphology. Angiogenesis, tumour development and tissue regeneration.	Deafness. Hepatocellular carcinoma.	+	+
VCAN	versican	Large proteoglycan ECM molecule. Cell adhesion, differentiation, proliferation and migration.	Wagner syndrome.	+	+
C3	complement C3	Complement activation, inflammation, immunity, neural development.	Haemolytic Uremic syndrome.		

1 Gene symbol based on HUGO Gene Nomenclature Committee (HGNC), accessible at http://www.genenames.org/
2 Function based on OMIM gene description, accessible at http://www.omim.org/
3 Associations based on GeneCards human gene database, accessible at http://genecards.org/

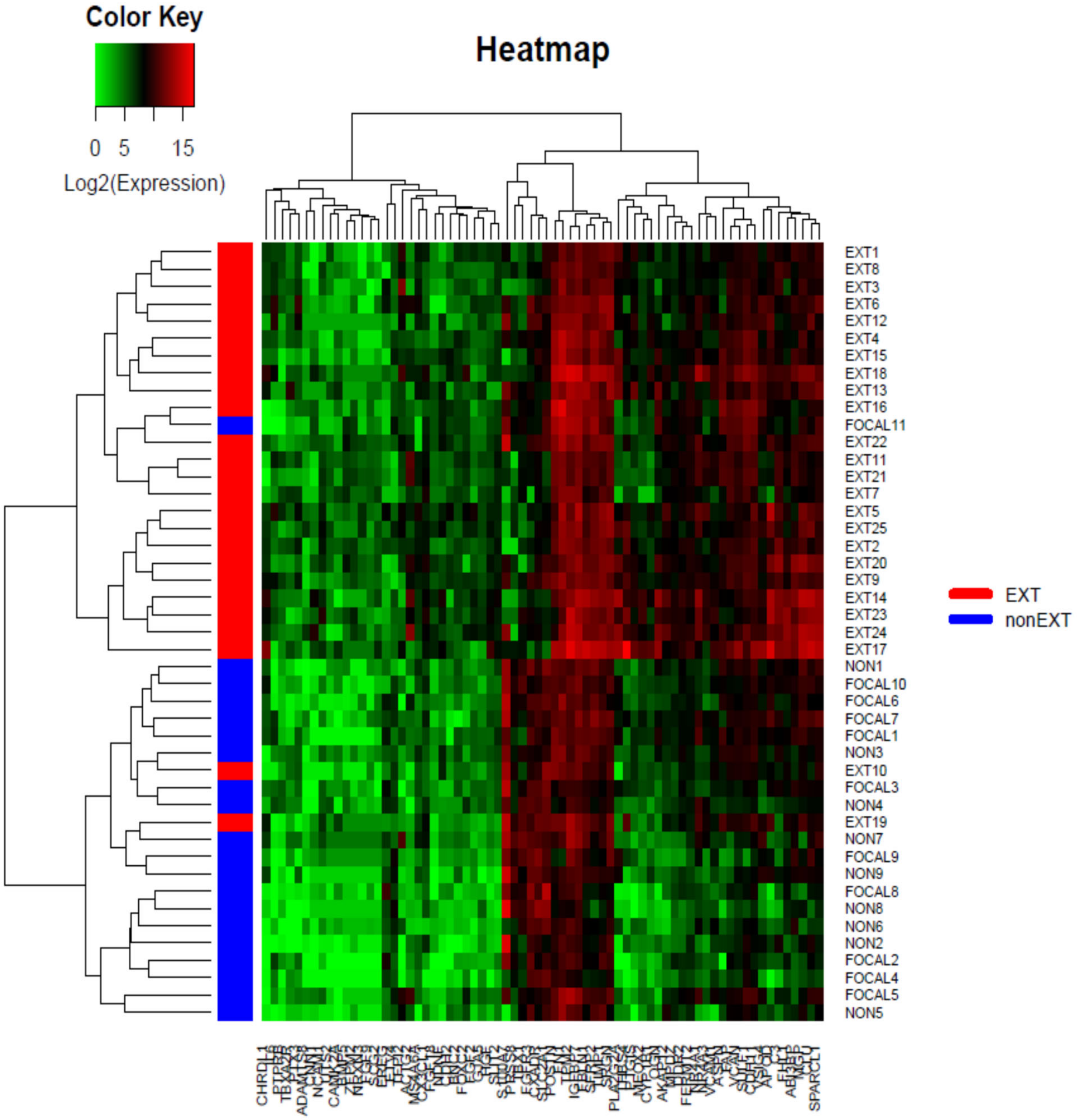

Supplementary Figure 1. Heatmap of the normalized data, scaled to give all genes equal variance, generated via unsupervised clustering based on the highest ranking differentially expressed genes between EXT (red) and Focal/Non-PNI (blue – nonEXT) cohorts with fold change >3, < -3 and adjusted *P*-value < 0.01. Green represents low expression, Red represents high expression.
